# Global stakeholder preferences for point-of-care HIV nucleic acid amplification tests: A conjoint study

**DOI:** 10.1371/journal.pone.0361025

**Published:** 2026-10-22

**Authors:** Kara M. Palamountain, Chethana Achar, Rebecca P. Kirby, Yukari C. Manabe, Debi Boeras, Michal Maimaran

**Affiliations:** 1 Kellogg School of Management, Northwestern University, Evanston, Illinois, United States of America; 2 Division of Infectious Diseases, Department of Medicine, Johns Hopkins University School of Medicine, Baltimore, Maryland, United States of America; 3 Global Health Impact Group, Atlanta, Georgia, United States of America; Oregon State University, UNITED STATES OF AMERICA

## Abstract

**Introduction:**

This study aimed to assess global stakeholder preferences for key attributes of point-of-care (POC) HIV Nucleic Acid Amplification Tests (NAAT), with the aim of informing product development to support progress toward UNAIDS 95-95-95 targets.

**Methods:**

A web-based conjoint survey was conducted in August through November 2024 among HIV clinicians, laboratory professionals, and researchers. Participants were asked to evaluate hypothetical POC HIV NAAT profiles varying by six attributes: sensitivity / limit of detection (LOD), sample-collection method, turnaround time (TAT), result format, price, and power source. Relative importance and part-worth utilities were calculated to assess trade-offs in decision-making.

**Results:**

One hundred three respondents from 33 countries, primarily in Africa (75%), completed the survey. The relative importance placed on the attributes in the conjoint task varied based on the setting. In community and home-based settings, participants prioritized fingerstick sample collection (25.90%) and faster TATs (28.8%), even at the expense of sensitivity (15.70%). By contrast, respondents from hospitals and health centers valued tests with high sensitivity (42.6%, 40.9%) and faster TATs (31.5%, 22.6%). Across all respondents, price (7.0%), result format (3.30%), and power source (7.25%) were less influential.

**Conclusions:**

Stakeholders were willing to make trade-offs among desirable attributes, prioritizing rapid turnaround time, strong sensitivity, and fingerstick sampling over affordability, power requirements, and result format in the conjoint task. These preferences vary by level of care and by use case, underscoring the importance of aligning product features with contextual needs. Engaging stakeholders early in the design process is critical to ensure new POC HIV NAAT technologies are both acceptable and scalable in low-resource settings.

## Introduction

As of 2023, an estimated 39.9 million people are living with HIV globally [1]. Although significant progress has been made in expanding access to antiretroviral therapy (ART), gaps in diagnosis, treatment adherence, and monitoring persist [2]. In response, UNAIDS launched the “95-95-95” global targets; by 2025, 95% of people living with HIV will know their HIV status (The First 95), 95% of those diagnosed will receive sustained ART (The Second 95), and 95% of those on ART will achieve viral suppression (The Third 95) [2].

Achieving these targets, which as of 2024 stood at 87-88-95, respectively, requires not only access to treatment but also effective tests (Table 1) across the care cascade [2,3]. This begins with serologic testing, primarily antibody/antigen rapid diagnostic tests, which remain the primary method for achieving the First 95 target [4,5]. However, nucleic acid amplification tests (NAATs) are essential in specific contexts such as early infant diagnosis [4] and during PrEP initiation, where serologic tests may be unreliable [4–8]. A quantitative NAAT is also critical for treatment initiation to assess baseline viral levels and ongoing monitoring to track treatment success [9–13]. WHO defines viral suppression as <1,000 RNA copies per milliliter (mL), whereas the U.S. CDC uses a more conservative threshold of <200 RNA copies per mL [9,10].

**Table 1:** HIV Care Cascade Testing Table.

Stage of HIV Care Cascade	Role of NAAT	Role of Serologic Testing
First 95 – Diagnosis	Used in early infant diagnosis [4] and for detecting acute HIV infection before PrEP initiation; essential when serologic tests may be unreliable [4–8].	Primary tool for diagnosis using HIV antibody/antigen combination assays; widely accessible via RDTs [4,5].
Second 95 – Treatment Initiation	Establishes baseline viral load prior to ART, serving as a reference for evaluating treatment response [9].	Not typically used at this stage.
Third 95 – Viral Suppression	Gold standard for monitoring ART efficacy; detects treatment failure and informs clinical decisions. WHO defines suppression as <1,000 copies per mL; CDC uses <200 copies per mL [9–13].	Not applicable; viral load testing is the standard for monitoring.

Note: HIV nucleic acid amplification tests (NAATs) include both qualitative and quantitative assays that are often—but not always—performed on the same instruments. Qualitative HIV NAATs detect the presence of HIV RNA or proviral DNA and provide a binary result (positive / negative) with reliable detection above the Limit of Detection (LOD). Because detection of proviral DNA is acceptable for diagnostic purposes, these tests can use various sample types—such as whole blood or dried blood spots—without necessarily requiring plasma. By contrast, quantitative HIV RNA NAATs require plasma to avoid interference from proviral DNA, which would confound accurate measurement of active viral replication. These tests yield numerical results in copies per millilitre, reliably above the Limit of Quantification (LOQ); linearity and precision are additional performance characteristics relevant to quantitative test accuracy.

### Access and the need for POC innovation

Despite the importance of NAATs, access remains a challenge, particularly in low-resource or decentralized settings. Conventional NAAT depends on centralized laboratories, skilled personnel, and stable infrastructure [12]. Near point-of-care (POC) platforms have improved access and reduced TATs, but as Drain et al. (2019) noted, they are still dependent on laboratory infrastructure (e.g., centrifugation) and are not truly POC [12].

WHO originally proposed the ASSURED criteria—Affordable, Sensitive, Specific, User-friendly, Rapid, Equipment-free, and Deliverable—to guide the development of diagnostics, particularly lateral flow assays, for use in resource-limited settings. In 2018, these criteria were expanded to REASSURED, incorporating Real-time connectivity and Ease of specimen collection [14–16]. Multiple groups have since developed target product profiles (TPPs) outlining ideal POC HIV quantitative NAAT characteristics (see Table 2) [12, 17, 18, S. Mulvaney, personal communication, June 27, 2025]. These profiles reflect a consistent push for sensitive, rapid, user-friendly solutions suited to low-resource environments.

**Table 2:** Comparison of Target Product Profiles (TPPs) for POC HIV NAAT.

Attribute	2013 IDC Draft TPP [17]	2019 Drain et al.[12]	2024 RADx Program[18, S. Mulvaney, personal communication, June 27, 2025]
Goal	Quantification of HIV RNA copies per mL	Quantification of HIV RNA copies per mL	Quantification of HIV RNA copies per mL
LOD	≤1,000 copies per mL	≤200 copies per mL	1,000 copies per mL or better (e.g., 200 copies per mL)
Precision	Within ±0.5 log_10_ copies per mL compared to standard laboratory methods	Within ±0.3 log_10_ copies per mL compared to reference gold standard test	Not specified
TAT	≤90 minutes	≤30 minutes	≤60 minutes, but ideally ≤30 minutes
Throughput	5–10 tests per 8-hour day	Minimum of 15 tests per 8-hour workday	Not specified
Sample Type	Whole blood, plasma, or serum	Finger capillary whole blood (maximum, 200 μl) or heel stick for young children	Utilizes sample volumes compatible with POC collection
Sample Volume	≤200 µL	200 µL	Employed at the POC setting and preferably CLIA-waived, but on- or off-cartridge processing and detection out of plasma or serum
Equipment Requirements	Portable, robust, operable in various environmental conditions; minimal reliance on external power sources	Small, portable, robust; uninterrupted power supply; functional across a wide range of temperatures	Ability of the solution to serve/deploy to underserved, hardly reached communities is highly desirable
Ease of Use	Operable by minimally trained healthcare workers; minimal maintenance	Operable by local healthcare workers with minimal training; automated processes	Employed at the POC setting and potentially CLIA-waived
Result Format	Quantitative output	Quantitative output	Not specified
Data Management	Capability to store and transmit data for patient management and surveillance	Results should be archivable to a centralized management system for monitoring test volumes, results, and error rates	Not specified
Cost per Test	Affordable for resource-limited settings; specific target not specified	Affordable to resource-limited HIV programs funded by governments and global donors; specific target not specified	Not specified; Competitive with alternatives and appropriate for POC

Ultimately, the development of feasible tests requires trade-offs. No single test can maximize every desirable feature at once, so developers must balance LOD, sample type, TAT, result format, cost, and operational needs against one another. Stakeholder input is essential to ensure tests meet real-world expectations and constraints. To understand the preferences of diverse stakeholders, we employed a conjoint study (sometimes referred to as “trade-off analysis”), which measures the relative value stakeholders assign to individual product features [19–22].

Conjoint analysis works by asking respondents to evaluate a series of hypothetical product profiles, each a different bundle of feature levels. For example, one profile might pair a low price with a slow TAT, while another profile might pair a high price with a fast TAT. Because respondents rate whole profiles rather than rating features in isolation, their choices reveal the trade-offs they are willing to make. We then use a regression analysis to work backward from these ratings and estimate a “utility” for each feature, a number presenting how much that feature drives respondents’ overall preferences. A higher utility means respondents value that feature more and are more willing to accept a less desirable level of another feature to obtain it.

## Methodology

This study consisted of an internet-based survey that was reviewed by Northwestern University IRB (STU00220919) and was determined to meet the criteria for exemption. Patients and/or the public were not involved in this study, as the research focused on stakeholder preferences related to diagnostic test attributes rather than direct patient care or patient-reported outcomes. We conducted a web-based conjoint survey among HIV clinicians, laboratory professionals, researchers, and implementation scientists. The survey was open from August 14 to November 22, 2024. Respondents were recruited through professional networks and listservs, including the Clinton Health Access Initiative, FIND, UNAIDS, and the Global Health Impact Group. A probability sample was not possible, given that no sampling frame exists for this population. We encouraged snowballing by asking respondents to distribute the survey with colleagues. Participants were not compensated. Eligible participants provided informed consent electronically.

### Survey content

The survey collected demographic information (organization type, role, gender, and geography), current access to HIV NAAT platforms, primary use case (initial diagnosis, initiating ART/monitoring on treatment) and where new POC tests should be prioritized with the healthcare system (Community or Home, Health centers, District hospital or above). Respondents were asked to identify the primary barrier that a new test should address. Full survey instrument is provided (S1 File).

The main portion of the survey consisted of the conjoint task designed to estimate the utilities and relative importance of the following six attributes with 14 attribute levels (Table 3).

**Table 3:** Attributes and Levels for POC HIV NAAT Conjoint.

Attribute	Attribute Description	Attribute Level
LOD	Lowest number of copies of HIV RNA virus detected per mL of plasma	200 copies / mL
		1000 copies / mL
Sample	How test sample is collected	Fingerstick
		Venipuncture
Power	How the test device is powered	Mains power with electricity
		Mains power with battery backup up to 12 tests
Price	Price of the test	$8
		$10
		$12
Result	Semi-quantitative (SQ) at LOD (e.g., > 1000 copies per mL) or quantitative (Q) (e.g., 1243 copies per mL)	Semi-quantitative
		Quantitative
TAT	Time, in minutes, from sample collection to result	15 minutes
		30 minutes
		60 minutes

We selected these attributes and attribute levels based on the existing POC NAAT TPPs (Table 2) as well as qualitative research conducted among stakeholders. A detailed discussion of this process of selecting attributes and attribute levels is provided (S2 File). In several cases, the range of attribute levels extends from levels achievable by current or near-term tests to levels that represent aspirational targets not yet met by any test; we took this approach deliberately, since TPPs are themselves intended to guide future test development rather than describe only existing tests, and a conjoint design restricted to currently achievable levels would understate stakeholder demand for performance improvements.

Combining these 14 attribute levels yields 144 (2 × 2 × 2 × 3 × 2 × 3) possible test profiles. Presenting all 144 to each respondent is infeasible and unnecessary, because conjoint analysis can estimate the utility of every attribute level from a carefully chosen subset of profiles and then recombine those utilities to predict the preferences for and of the 144 combinations [19–22].

Using the AlgDesign package in R [23], we selected a subset of 12 profiles from the 144 possible combinations. Profiles were chosen so that attribute levels were balanced across the design and varied as independently of one another as possible, the condition required to estimate each attribute's utility without confounding. Each level of every attribute appeared equally often (each binary level in 6 of 12 profiles; each of the three price and TAT levels in 4 of 12 profiles). The resulting design was highly efficient (D-efficiency = 0.99, where 1.0 denotes a perfectly orthogonal design) and near-orthogonal: all pairwise correlations among the six attributes were zero except for a small correlation between price and TAT (r = −0.16), the mean absolute inter-attribute correlation was 0.01, and all variance inflation factors were at or near 1.0 (maximum 1.03), indicating negligible multicollinearity [24].

Participants were asked to rate the 12 hypothetical HIV test concepts, each representing a unique combination of the attribute levels, on a 7-point scale (1 = Not Likely to purchase/use/recommend, 7 = Very Likely to purchase/use/recommend) for their prioritized use case and level of the health system they indicated in the first part of the survey. The presentation order of 12 test concepts was randomized to reduce order effects.

## Results

A total of 278 individuals accessed the online survey and 103 completed the survey, corresponding to a response rate of approximately 37%. These 103 respondents were from 33 countries and they completed the survey (S4 File) with an average completion time of 11 minutes. Of these respondents, 36% (n = 37) identified as female, 62% as male (n = 64), and 2% (n = 2) as Other. Of these respondents, 74.8% (n = 77) were from Africa, with Nigeria, Kenya, and Uganda as the top contributors. Smaller proportions came from the Western Pacific 10.7% (n = 11), the Americas 7.8% (n = 8), South-East Asia 3.9% (n = 4), and the Eastern Mediterranean region 2.9% (n = 3).

Of the 103 respondents, 51.5% (n = 53) were categorized as non-laboratory professionals, including academic researchers 28.3% (n = 15) and program managers 24.5% (n = 13) (S5 File). The remaining 48.5% (n = 50) were laboratory professionals (technicians 62% (n = 31) and directors 38% (n = 19)).

Preferred Use Case: When asked to prioritize the use case for new POC NAAT, 56.31% selected initial diagnosis (n = 58), and 41.75% (n = 43) selected initiating / monitoring, and 1.94% selected Other (n = 2).

Preferred Healthcare Levels: When asked to prioritize where new POC NAAT should be implemented, 40.78% (n = 42) selected health centers, 35.95% (n = 36) preferred district hospitals or higher-level facilities, 22.33% (n = 23) prioritized community or home-based settings, and 1.94% (n = 2) selected Other.

### Attribute importance

Following the standard conjoint analysis, we used linear regression modeling to analyze the impact of each attribute on participants’ likelihood of adopting the test [25]. Models were stratified by preferred use case, healthcare level, geography, and laboratory role to detect variations in preferences by setting.

Using the regression results, we calculated importance weights for each attribute. Importance weight refers to how much influence a particular attribute has on participants’ overall preferences, relative to other attributes. The greater the number, the more that attribute contributed to participants’ ratings. Table 4 summarizes Importance Weights overall as well as by role, geography, preferred use case, and preferred health-system level.

**Table 4:** Attribute Importance Weights.

Attribute	Respondent Type									
	Entire sample	Role		Geography*		Preferred Use Case for POC NAAT**		Preferred Level of Health System for POC NAAT**		
	*All N=103*	*Lab N=50*	*Non-Lab N=53*	*Africa N=77*	*Non- Africa N=26*	*Initial Diagnosis N=58*	*Initiating / Monitoring N=43*	*Hospital or Higher N=36*	*Health Centers N=42*	*Community/Home N=23*
LOD	**36.50%**	**37.60%**	**31.10%**	**35.80%**	**33.80%**	**36.10%**	**36.30%**	**42.60%**	**40.90%**	**15.70%**
Sample	**17.30%**	12.90%	**18.10%**	**16.20%**	**17.90%**	9.90%	**26.70%**	5.20%	14.70%	**25.90%**
Power	7.20%	7.30%	6.20%	10.90%	2.50%	5.10%	6.90%	2.70%	10.10%	10.50%
Price	7.00%	12.90%	13.80%	6.20%	14.50%	5.20%	8.80%	14.40%	7.60%	12.70%
Result	3.30%	0.70%	5.60%	0.20%	10.50%	0.80%	11.10%	3.60%	4.10%	6.50%
TAT	**28.70%**	**28.70%**	**25.10%**	**30.70%**	**20.80%**	**42.90%**	10.10%	**31.50%**	**22.60%**	**28.80%**

Note: Numbers within cells represent importance weights, reflecting the influence of each attribute relative to other attributes on respondent preferences. Bolded numbers are statistically significantly different from 0 at p < 0.05.

* We compare respondents from Africa and non-Africa locations because 75% of the sample (N = 77) were from Africa, and the rest from various locations.

** Sample size for Preferred Use Case and Preferred Level of Health System were 101 because two respondents who selected “Other” were excluded.

Overall Importance: Across all levels of care, the most influential attributes in test adoption were (in order) the LOD (36.5% importance), TAT (28.7% importance), and sample-collection method (17.3% importance). Attributes such as price, result format, and power source were considered less critical (not statistically significant; p > 0.05) in influencing adoption decisions.

Importance by Role: We also analyzed if preferences varied between Laboratory and non-Laboratory respondents. Results showed Laboratory respondents valued LOD (37.6% importance) and TAT (28.7% importance) only, whereas Non-Laboratory respondents valued LOD (31.1% importance), TAT (25.1% importance), and sample collection (18.1% importance). Other attributes—price, result format, and power—were not important (statistically not significant at p < 0.05) across all roles.

Importance by Geography: We also analyzed if importance weights varied between Africa and Non-Africa respondents. Results showed respondents based in Africa value LOD (35.8% importance), TAT (30.7% importance), and sample-collection method (16.2% importance). For non-African respondents, the same attributes were important but varied in the weightage of importance. Non-African respondents indicated choices that suggest they valued LOD (33.8% importance), TAT (20.8% importance), and sample-collection method (17.9% importance). Notably, TAT was more important to the African respondents. Other attributes—price, result format, and power—were not important (statistically not significant at p < 0.05) across all respondents.

Importance by Use Case Level: We also analyzed if importance weights varied between use cases prioritized by the survey respondents for POC NAAT. Results showed when initial diagnosis was prioritized, respondents valued LOD (36.1% importance) and TAT (42.9% importance) only, however, when initiating/monitoring was prioritized, respondents valued LOD (36.3% importance) and sample collection (26.7% importance). Other attributes—price, result format, and power—were not important (statistically not significant at p < 0.05) across all use-case levels.

Importance by Health-System Level: We also analyzed if importance weights varied between health-system levels prioritized by the survey respondents for POC NAAT. At health centers and hospitals, LOD was the most important factor, followed by TAT. By contrast, at community or home, respondents placed the highest value on TAT (28.8% importance) and sample-collection method (25.9%), with LOD still considered important (15.7%) but less so than in other clinical settings. Consistent with the previous analyses, price, result format, and power were not important (statistically not significant at p < 0.05) across all health-system levels.

### Attribute-level utilities

Next, we estimated part-worth (PW) utility coefficients. PWs are calculated from the linear regression described in the “Attribute Importance” section. PWs reflect the relative value participants assign to each level within an attribute—higher values indicate greater preference, whereas lower values signal lower desirability. These estimates reveal which attribute levels are driving preferences within each category.

Table 5 shows the average PWs for each attribute across the entire sample as well as by role, geography, preferred use case, and preferred level of healthcare system. A PW utility of zero denotes the reference (omitted) level for each attribute (200 copies per mL, fingerstick, battery backup, $8, quantitative, and 15 minutes). These levels are selected to be the reference levels arbitrarily. Changing the reference levels does not change the results.

**Table 5:** Part-Worth (PW) Utility Coefficients.

Attribute	Attribute Levels	Respondent Type					Preferred Use Case for POC NAAT		Preferred Level of Health System for POC NAAT		
		Entire sample	Role		Geography						
		*All N = 103*	*Lab N = 50*	*Non-Lab N = 53*	*Africa N = 77*	*Non- Africa N = 26*	*Initial Diagnosis N = 58*	*Initiating / Monitoring N = 43*	*Hospital or Higher N = 36*	*Health Centers N = 42*	*Community/Home N = 23*
**LOD**	200 copies per mL	**0.000**	**0.000**	**0.000**	**0.000**	**0.000**	**0.000**	**0.000**	**0.000**	**0.000**	**0.000**
	1000 copies per mL	−0.777	−0.744	−0.872	−0.721	−0.829	−0.635	−0.910	−0.811	−0.909	−0.469
**Sample**	fingerstick	**0.000**	**0.000**	**0.000**	**0.000**	**0.000**	**0.000**	**0.000**	**0.000**	**0.000**	**0.000**
	venipuncture	−0.369	−0.337	−0.462	−0.247	−0.483	−0.175	−0.670	−0.098	−0.379	−0.774
**Power**	mains power with electricity	−0.152	−0.225	**0.064**	−0.139	−0.164	−0.089	−0.253	**0.052**	−0.235	−0.313
	mains power with battery backup up to 12 tests	**0.000**	**0.000**	0.000	**0.000**	**0.000**	**0.000**	**0.000**	0.000	**0.000**	**0.000**
**Price**	$8	0.000	0.000	**0.000**	0.000	**0.000**	0.000	0.000	0.000	0.000	**0.000**
	$10	**0.007**	**0.068**	−0.172	**0.248**	−0.219	**0.005**	**0.041**	**0.142**	**0.099**	−0.378
	$12	−0.141	−0.062	−0.375	0.100	−0.368	−0.086	−0.180	−0.132	−0.148	−0.141
**Result**	semi-quantitative	−0.071	−0.004	−0.270	**0.013**	−0.150	−0.014	−0.174	**0.069**	−0.121	−0.194
	quantitative	**0.000**	**0.000**	**0.000**	0.000	**0.000**	**0.000**	**0.000**	0.000	**0.000**	**0.000**
**TAT**	15 minutes	**0.000**	**0.000**	**0.000**	**0.000**	**0.000**	**0.000**	**0.000**	**0.000**	**0.000**	**0.000**
	30 minutes	−0.321	−0.405	−0.072	−0.266	−0.373	−0.428	−0.122	−0.289	−0.264	−0.48
	60 minutes	−0.611	−0.637	−0.535	−0.549	−0.670	−0.755	−0.279	−0.599	−0.491	−0.861

In addition to relative preference, PW analysis allows us to identify the ideal test. The bolded values in Table 5 indicate the most preferred level within each attribute, that is, the level with the highest estimated PW. Across most groups, the ideal test is composed on 200 copies per mL, fingerstick sample, main power with battery backup, costs $8, quantitative results, and TAT of 15 minutes. Manufacturing the ideal test may not be possible due to budget or feasibility constraints, but the PWs analysis allows us to identify which products would be considered alternatives, as we elaborate in the Discussion section.

## Discussion

Across all respondent groups, the highest-scoring test design included the following attributes: an LOD of 200 copies per mL, a cost of $10 per test (though some groups have higher PW to the $8 test), fingerstick sample collection, a 15-minute TAT, battery backup (though some groups have higher PW to the mains power with electricity test), and a quantitative-result format (though some groups have higher PW to the semi-quantitative test).

Although this combination represents the highest theoretical preference, such a test may not be technically feasible. To achieve a 200 copies per mL detection limit using less than 200 μL of capillary blood (which would be the case using the preferred fingerstick test), product developers will need breakthrough innovation in sample preparation and amplification [12]. In such cases, test developers face a trade-off—whether to prioritize lower detection thresholds that require venipuncture or to accept a higher LOD in exchange for easier fingerstick sampling.

Overall, a venipuncture test with an LOD of 200 copies per mL has a combined utility of 0 + (−0.369) = −0.369, which is higher than a fingerstick test with an LOD of 1,000 copies per mL −0.777 + 0 = (−0.777), assuming all other attribute levels are the same across the two tests. But these trade-offs may vary across respondent groups and preferred use cases. For example, at the home/community level (level 0), stakeholders expressed a preference for a test with a fingerstick sample (PW utility = 0) and an LOD of 1,000 copies per mL (utility = –0.469), resulting in a combined utility of 0+ (−0.469) = –0.469, over a venipuncture-based test (utility = –0.774) with an LOD of 200 copies per mL (part-worth utility = 0), which had a lower combined utility of 0+ (−0.774) = –0.774. This finding suggests ease of sample collection may outweigh analytical sensitivity in lower-level care settings. Note only two LOD values—200 and 1,000 copies per mL—were evaluated in this study, in line with WHO and US CDC thresholds.

TAT emerged as the second most important attribute across most respondent groups, according to the calculated importance weights. Notably, at the home/community level, TAT was identified as the most important attribute overall and was also the most important attribute when initial diagnosis was selected as the preferred use case for POC HIV NAAT.

Although a 15-minute turnaround is ideal, turnaround times for existing commercially available or late-stage POC HIV NAAT assays range from 55 to 90 minutes [12]. Increasing the sample volume via venipuncture could theoretically reduce processing time, by delivering more viral particles into the reaction, requiring fewer amplification cycles to reach a detectable signal. In practice, however, this theoretical gain is often offset because venipuncture-collected samples are conventionally processed as plasma, which require a separate centrifugation step to separate plasma from whole blood, adding 15–20 minutes to overall time to result [12]. Similarly, a fingerstick sample despite its smaller volume, will also require some method of plasma conversion to generate a quantitative, plasma-equivalent result, which, may likewise add processing time. Because both sample types are therefore subject to a time penalty from plasma separation whether performed off-device via centrifugtation or on-device via some other method, testing the impact of this added time is relevant regardless of sample collection method. At the home/community level, a 30-minute TAT with fingerstick (PW utility –0.48 + 0.00 = –0.48) is preferred to a 15-minute TAT with the use of venipuncture (utility 0.00 + –0.774 = –0.774). However, a 60-minute TAT with fingerstick (utility –0.861 + 0.00 = –0.861) is not preferred to the 15-minute TAT with venipuncture (utility 0.00 + –0.774 = –0.774), indicating extended wait times may significantly reduce the utility of a test in community-based settings.

These findings suggest that although moderate increases in turnaround time may be tolerated, exceeding a threshold—particularly in low-resource, patient-facing contexts—can lead to sharp declines in perceived value. This threshold effect illustrates why our conjoint levels were designed to span the feasible-to-aspirational range rather than only currently achievable specifications: doing so allowed us to identify the point at which incremental delays begin to substantially erode perceived value, a finding with direct relevance to near-term product engineering even though the underlying attribute levels included aspirational targets.

Surprisingly, price did not rank among the top three most important attributes for any respondent group in the conjoint analysis. This finding contrasts with participants’ direct responses in the survey, where 30% identified affordability as the primary barrier that new POC HIV NAAT should address. However, in the conjoint analysis, price emerged as the second-least-important attribute of the six evaluated. The narrow pricing range ($8-$12) may have limited its influence in the conjoint task. One other explanation is that the survey respondents may not be the direct payors for the test. In addition, the price attribute reflected only the per-test cost and did not include associated capital investments in equipment, which may be the true drivers behind affordability concerns reported in direct evaluation of barriers in the pre-conjoint questions.

The discrepancy may also reflect a broader methodological distinction between the forced-trade-off structure of conjoint analysis and the unconstrained format of the direct barrier-identification question. In the conjoint task, price was evaluated in relation to the other five attributes within a fixed set of profiles. This forced trade-off approach revealed that price was less important relevant to LOD and TAT that captured a large share of relative importance across nearly all subgroups. By contrast, the direct barrier-identification question asked about affordability independent of any trade-off, allowing it to register as a leading concern without being weighed against competing product characteristics. We therefore interpret the low conjoint-derived importance of price as reflecting its relative priority against highly salient clinical attributes within this specific trade-off structure, rather than evidence of affordability is not a genuine barrier to adoption.

### Study limitations and directions for future research

Given the relatively low importance assigned to attributes such as power source and result format, future conjoint analyses may consider removing these attributes and expanding the range of LOD levels (e.g., 40 or 500 copies per mL) to better reflect the competitive landscape of near POC HIV NAAT technologies. Similarly, a broader range of price points should be explored to capture more realistic thresholds for acceptability and rejection. This ability would allow product developers to better understand market-specific boundaries for pricing and ensure affordability targets aligned with stakeholder expectations across different settings.

Importantly, the value assigned to price in the current study may have been influenced by the limited pricing range presented ($8–$12) and may not reflect current stakeholder concerns given recent shifts in the global funding landscape between October 2024 and July 2026.

The survey included only semi-quantitative and quantitative result types, omitting a qualitative option. Including a qualitative result type may have been beneficial, because most respondents prioritized initial diagnosis as the primary use case for POC HIV NAAT. Given that current tests used for initial diagnosis are typically qualitative, respondents may have been willing to accept a qualitative result—particularly if it improved the LOD or diagnostic accuracy.

Several attribute levels in our survey combined related but potentially distinct use cases or levels of the health system, which may obscure meaningful differences in stakeholder preferences. First, the survey grouped ART initiation and treatment monitoring into a single use-case category; however, these represent clinically distinct moments in the care cascade. Specifically, a one-time decision at treatment start versus ongoing longitudinal surveillance, and stakeholder preferences may differ meaningfully between those use cases. We considered further disaggregating use cases during survey design, including separating PrEP initiation from PrEP monitoring, but ultimately grouped related use cases together to preserve statistical power per subgroup. Relatedly, the survey grouped several initial diagnosis use cases together (e.g., screening in newborns; replacement of antibody/antigen testing in adults; recent HIV exposure; adults on pre-exposure prophylaxis), and preferences may also vary within these subcategories. Second, the survey grouped home-based and community-based testing into a single level within the level of the health system question. These settings likely vary in important ways that our data cannot capture. For instance, home-based testing may be more likely to rely on power sources independent of mains electricity. Additionally, we did not test alternative levels for power configuration, an omission that is notable given the emphasis the REASSURED criteria and related TPPs position on mains-independent power. Future work could explore these distinctions.

Although the respondent pool was geographically and professionally diverse, many sub-categories had limited representation. Non-laboratory roles were dispersed across small subgroups, and no individual country contributed more than 12 respondents, limiting meaningful comparisons across specific roles or geographies. To enable a more robust analysis, we grouped respondents into broader categories (e.g., laboratory vs. non-laboratory roles, Africa vs. non-Africa respondents), but findings at this level should be interpreted with caution. These limitations highlight that results are preliminary and hypothesis-generating. Larger, more targeted surveys are needed to validate these findings and further refine value propositions for POC HIV NAAT technologies.

Researchers and laboratory personnel dominated the survey respondents, with very few clinicians and no patient respondents. Consequently, attributes such as turnaround time, ease of use, and workflow integration may not fully reflect the priorities of clinicians working at the point of care or the patients who ultimately experience the benefits and burdens of testing.

Moreover, the survey did not capture key contextual details such as the respondents’ involvement in HIV NAAT procurement or financing, or how HIV NAAT integrates into broader diagnostic pathways (e.g., rapid diagnostic or self-testing). If respondents did not have direct experience in procurement, financing decisions, or day-to-day operational challenges, attributes such as cost, result output format, and power requirement may have been undervalued. These same caveats also apply to the higher weights attributed to LOD and TAT: these preferences largely reflect the perspectives of non-clinicians and non-patients, and may not represent the priorities of those making POC decisions or directly managing patient care. Supplemental qualitative research could help clarify how these attributes are prioritized by clinicians, procurement officers, and end users in real-world settings, particularly in lower-level or community facilities, and provide insight into why power requirements were not viewed as more significant or how users perceive the practical value of a 15-minute versus one-hour TAT (e.g., whether shorter TAT improves clinic throughput or reduces patient engagement time).

Regional context may also shape preferences. In our survey, more than two-thirds of respondents were from Africa—where HIV burden is highest—and many prioritized initial diagnosis, likely reflecting needs around early infant diagnosis [4]. While attribute preferences between use cases did not vary in this survey, preferences in other regions may differ, for example in geographies placing greater emphasis on PrEP initiation or treatment monitoring. Additional geographically focused conjoint studies could help clarify these regional differences. Furthermore, such research could explore which use cases respondents have in mind when considering patients with viral loads between 200 and 1,000 copies per mL. Further stakeholder education may be necessary to help users understand the true clinical significance of the tradeoff between 200 and 1,000 copies per mL LOD. Although stakeholders in this conjoint consistently prioritized 200 copies per mL, a 1,000 copies per mL LOD may offer sufficient sensitivity in many contexts, for example, not only for initiating and monitoring HIV viral load, but also in PrEP and diagnostic algorithms supporting HIV elimination.

Taken together, these limitations indicate that the findings primarily reflect the perspectives of laboratory and research stakeholders. Future surveys should include deeper sampling of clinicians and, where relevant, patient groups, as well as more detailed contextual information to ensure that TPP attributes align with the needs and priorities of all key user communities.

## Conclusion

This study provides key insights into stakeholder preferences for POC NAAT attributes. Preferences vary by healthcare level, where sensitivity (low LOD) and speed were prioritized for clinical settings, while ease of sample collection and rapid results were prioritized for community-level settings. Preferences also varied by intended use case. Respondents who identified initial diagnosis as the primary use case for POC HIV NAAT prioritized speed over sensitivity (low LOD), while those selecting initiation and treatment monitoring as the primary use case favored sensitivity (low LOD) and ease of sample collection over speed.

Overall, these findings suggest stakeholders are willing to compromise on cost, power, and result format but expect tests to be sensitive, fast, and easy to use—particularly in community-based contexts. This should not be interpreted as evidence that affordability is not a genuine concern: the discrepancy with the direct barrier-identification question, in which affordability was the most commonly cited barrier, suggests that price remains a critical consideration in absolute terms even when it is deprioritized in relative, forced-trade-off comparisons with the prices reflected ($8, $10, $12). This result was surprising given the challenges that many communities and homes face with consistent and reliable power [26].

These findings can guide developers in making informed trade-offs when designing POC HIV NAAT and inform policymakers. Incorporating stakeholder input early in the development process increases the likelihood of adoption and successful implementation, particularly in low-resource or decentralized settings [27,28]. As the global health community pushes toward the 95-95-95 goals, ensuring equitable access to effective POC HIV NAAT tools across all levels of care is essential.

## Supporting information

S1 File Survey Questions. (PDF)

S2 File Attribute Selection. (DOCX)

S3 File Raw Data for 103 Respondents. (XLSX)

S4 File Survey Respondents by Region. (XLSX)

S5 File Survey Respondents by Lab Categorization / Role. (XLSX)
