# Supplementary material for: Global stakeholder preferences for point-of-care HIV nucleic acid amplification tests: A conjoint study
Source: PLoS One. 2026 Oct 22;21(10):e0361025. doi: 10.1371/journal.pone.0361025 (PMC13690244; doi:10.1371/journal.pone.0361025)
Supplement: Supplementary file 1 [file pone.0361025.s001.pdf]

Age

Are you **older than 21 years** of age?

- ○ Yes
- ○ No

unable

Thank you, but you do not qualify for this survey.

Introduction

Given your expertise, we would like to invite you to participate in this 10 minute survey to **better understand user sentiments around point-of-care testing for HIV viral load.**

Your feedback will provide guidance for the development of tests that aim to improve health outcomes by enabling early and accurate detection of HIV and timely initiation and monitoring of antiretroviral therapy. Your opinion is valued and we thank you for your time.

Please review the consent form here.

If you wish to participate, please click the “Yes, I consent to participate” button and you

will be taken to the survey. If you do not wish to participate in this study, please select "No, I do not consent to participate" or select X in the corner of your browser.

- ◯ Yes, I consent to participate
- ◯ No, I do not consent to participate

Demographic

1. In which country do you work primarily?

[▾]

2. Which gender identity best describes you?

- ◯ Male
- ◯ Female
- ◯ Non-binary / third gender

3. Which of the following best describes the organization where you work primarily?

- ◯ Academic Institution
- ◯ Advocacy Organization
- ◯ Consultant
- ◯ Funder / Donor
- ◯ Government / Ministry of Health
- ◯ Hospital or Clinic
- ◯ Industry
- ◯ Implementer

- ○ International Body
- ○ Laboratory
- ○ Non-governmental organization (NGO)
- ○ Technical Agency
- ○ [ ] Other

4. Which of the following best describes your role / activity within the organization where you work primarily?

- ○ Academic Researcher
- ○ Clinic Manager
- ○ Community Health Worker
- ○ Distribution / Supply chain
- ○ Doctor - Primary Care
- ○ Doctor - Specialist
- ○ Hospital Manager
- ○ Laboratory Director
- ○ Laboratory Technician
- ○ Manufacturing
- ○ Midwife
- ○ Nurse - Primary Care
- ○ Nurse - Specialist
- ○ Program Manager
- ○ Procurement
- ○ Technical Consultant
- ○ Product Developer / Innovator
- ○ Regulator
- ○ [ ] Other

Use Cases

5. Please indicate which "near" point-of-care (POC) HIV Molecular **Qualitative** tests are available to you currently (mark all that apply)

- ☐ Abbott m-PIMA
- ☐ Cepheid Xpert
- ☐ DRW Samba II
- ☐ Molbio Trueprep / Truelab
- ☐ [ ] Other
- ☐ None of the above

6. Please indicate which "near" point-of-care (POC) HIV Molecular **Quantitative** tests are available to you currently (mark all that apply)

- ☐ Abbott m-PIMA
- ☐ Cepheid Xpert
- ☐ DRW Samba II
- ☐ Molbio Trueprep / Truelab
- ☐ [ ] Other
- ☐ None of the above

7. If a **new** HIV Molecular **Quantitative** point-of-care (POC) test was to be developed,

what is the primary barrier it should address?

- ◯ Time to Result
- ◯ Sample Volume
- ◯ Sample Preparation Steps
- ◯ Sample Type
- ◯ Accuracy / Limit of Detection (LOD) or lowest number of copies of HIV RNA virus detected per mL of plasma
- ◯ Connectivity
- ◯ Other Steps Required
- ◯ Ability to withstand the operating and supply chain environment (temperature, humidity, etc.)
- ◯ Affordability
- ◯ Electricity requirements
- ◯ [ ] Other

Concept Testing

8. If a **new** HIV Molecular point-of-care (POC) test was to be developed, **what would be your primary or highest priority use case?**

Select one from list below:

- ◯ Initial diagnosis (e.g., Screening in Newborns ; Replacement of Antibody / Antigen Test in Adults ; Recent HIV Exposure ; Adults on Pre-exposure Prophylaxis (PrEP))
- ◯ Initiating/Monitoring treatment for people living with HIV
- ◯ [ ] Other

9. If a **new** HIV Molecular point-of-care (POC) test was to be developed, **what level of the healthcare system would you envision this test being used at?** Please identify which setting within your country or your national program would most apply?

- ◯ Community or Home (Level 0)
- ◯ Health centres (Level 1)
- ◯ District hospital or above (Level 2, 3, or 4)
- ◯ [ ] Other

10. To show that you are paying attention to the survey questions, please select 'Community or Home (Level 0)' option below.

- ◯ Community or Home (Level 0)
- ◯ Health centres (Level 1)
- ◯ District hospital or above (Level 2, 3, or 4)
- ◯ [ ] Other

11. Given your role as ${q://QID102/ChoiceGroup/SelectedChoices}, in ${q://QID79/ChoiceGroup/SelectedChoices}, imagine that you are looking to purchase/use/recommend an HIV Molecular point-of-care (POC) test for ${q://QID106/ChoiceGroup/SelectedChoices} in the ${q://QID121/ChoiceGroup/SelectedChoices} setting.

We will show you hypothetical products for such a test that vary across the following attributes:

1. ***Sample Collection Method:*** fingerstick (**FS**) or venipuncture (**V**)

2. ***Limit of Detection (LOD):*** lowest number of copies of HIV RNA virus detected per mL of plasma (**200** or **1000**)
3. ***Result Format:*** semi-quantitative (**SQ**) at LOD (e.g., > 1000 copies per mL) or quantitative (**Q**) (e.g., 1243 copies per mL)
4. ***Price:*** price of the test (**$8, $10, $12**)
5. ***Turnaround Time:*** time, in minutes, from sample collection to result (**15 min, 30 min, 60 min**)
6. ***Power Source:*** mains power with electricity (**Electricity**) or mains power with battery backup (**Battery**) for up to 12 tests

Please rate each of these products based on your likelihood of purchasing/using/recommending the test.

These products may not yet exist and we are only testing concepts.

	Not likely						Very likely
**FS** | **200** | **SQ** | **$12** | **15 min** | **Battery**	○	○	○	○	○	○	○
**V** | **200** | **SQ** | **$8** | **30 min** | **Electricity**	○	○	○	○	○	○	○
**V** | **1000** | **SQ** | **$12** | **30 min** | **Battery**	○	○	○	○	○	○	○
**FS** | **200** | **Q** | **$12** | **30 min** | **Electricity**	○	○	○	○	○	○	○

	Not likely						Very likely
**V** | **1000** | **SQ** | **$10** | **15 min** | **Electricity**	○	○	○	○	○	○	○
**FS** | **1000** | **SQ** | **$8** | **60 min** | **Battery**	○	○	○	○	○	○	○
**FS** | **200** | **SQ** | **$10** | **60 min** | **Electricity**	○	○	○	○	○	○	○

	Not likely						Very likely
**FS | 1000 | Q | $10 | 30 min | Battery**	○	○	○	○	○	○	○

	Not likely						Very likely
**V | 1000 | Q | $12 | 60 min | Electricity**	○	○	○	○	○	○	○
**FS | 1000 | Q | $8 | 15 min | Electricity**	○	○	○	○	○	○	○
**V | 200 | Q | $8 | 60 min | Battery**	○	○	○	○	○	○	○
**V | 200 | Q | $10 | 15 min | Battery**	○	○	○	○	○	○	○

Survey Completion

Thank you for completing this survey.

May we kindly request that you distribute the following survey link within your networks. https://kellogg.qualtrics.com/jfe/form/SV_8cb96xC3dOgUsdg.

Powered by Qualtrics
