# Supplementary material for: Global stakeholder preferences for point-of-care HIV nucleic acid amplification tests: A conjoint study
Source: PLoS One. 2026 Oct 22;21(10):e0361025. doi: 10.1371/journal.pone.0361025 (PMC13690244; doi:10.1371/journal.pone.0361025)
Supplement: Supplementary file 2 [file pone.0361025.s002.docx]

**S2 Appendix. Attribute Selection**

Attribute levels for the conjoint survey were selected to extend from levels achievable by current or near-term systems to levels that represent aspirational targets not yet met by any assay in development. We took this approach deliberately since TPPs are intended to guide future assay development rather than describe only existing products. A conjoint design restricted to currently achievable levels would not allow to identify stakeholders’ demand for performance improvements. Where the TPPs described a given attribute only qualitatively or vaguely, we supplemented these attributes with more quantitative or binary levels. Exact quantitative or binary values are required for the conjoint task, as we want to ensure all respondents understand the same level in the same way. Labelling price levels as, for example, “low, medium, or high” would allow subjective interpterion by different respondents. Giving exact numeric price levels mitigates this risk. These levels were informed by qualitative research in which we reviewed potential attributes and attribute levels with experts in HIV Viral Load (researchers, scientists, and clinicians) to identify which TPP-specified levels needed more definition or clarity for these stakeholders.

LOD is fundamentally constrained by sample volume, since a smaller input volume reduces the number of viral particles available for detection and raises the practically achievable limit of detection [12]. A test capable of meeting the WHO threshold (≤1,000 copies per mL) is more readily achievable using low-volume fingerstick sampling than one capable of meeting the more conservative CDC threshold (≤200 copies per mL), creating a direct trade-off between analytical sensitivity and ease of sample collection that we designed our conjoint LOD and sample-type attributes to probe.

This trade-off is defined at its two extremes by the sample attribute itself, which was set to fingerstick and venipuncture, reflecting the range of information provided around sample collection volume, method, and preparation described in the TPPs. At one end, plasma-based testing (as allowed for in the IDC TPP) was typically viewed by stakeholders a venipuncture sample collection method followed by centrifugation to separate plasma from whole blood, a laboratory-dependent workflow that falls outside most definitions of POC testing. At the other end, whole blood collected via fingerstick (as specified in the Drain TPP) was viewed by stakeholders as compatible with POC settings, but they noted that introduction of fingerstick whole blood presented a distinct technical challenge: because viral load is conventionally reported in copies per mL of plasma, generating a quantitative plasma-equivalent result from a whole blood input requires the device itself to perform some form of plasma separation, in addition to amplification and detection.

Power levels (mains power with electricity versus mains power with battery backup up to 12 tests) reflect the range of power and equipment requirements addressed only generally across the reviewed TPPs. One on end, the TPPs request *minimal reliance on external power sources* (as reflected in the IDC TPP). At the other end, the RADx TPP does not mention power at all. Stakeholders mentioned that power and backup power are often available at higher level facilities, and noted that installing a battery on a device would increase the price per test as a trade-off.

Price of the test ($8, $10, $12) was selected to reflect an anticipated future pricing trajectory for POC NAAT, informed by volume guarantee agreements in the broader viral load testing market [1]. Given the source TPPs describe affordability qualitatively rather than specifying price points, quantitative levels were necessary for the conjoint design.

Result format levels (quantitative, semi-quantitative) were informed by the IDC and Drain TPPs both of which specify a quantitative result format; the RADx TPP does not specify a result format and therefore did not inform level selection for this attribute. Semi-quantitative was included as a second level based upon stakeholder feedback, who noted that accepting a semi-quantitative result might enable favorable trade-offs, including faster TAT and simplified sample collection and processing.

TAT levels were set at 15, 30, and 60 minutes. Only one reviewed TPP (Drain) proposes an aggressive TAT of *≤* 30 minutes; 15 minutes was included as a level below this most stringent published target to characterize how stakeholder-perceived value continues to change beyond it. We note that 15 minutes falls below the range achievable by current or late-stage POC NAAT assays (approximately 55-90 minutes) [12], however, this level was included deliberately as an aspirational target rather than a near-term specification, a rationale we return to in the Discussion section. The more lenient 90-minute threshold specified in the 2013 IDC draft TPP was excluded because TATs for current commercially available and late-stage POC NAAT assays already range from 55 to 90 minutes; testing this older threshold would therefore have been unlikely to meaningfully differentiate stakeholder preferences from current baseline performance.

The published TPPs in Table 2 include additional characteristics beyond the six attributes tested here, such as throughput, ease of use, precision, and data management. Since six attributes resulted in 144 possible combinations, presenting the full set of TPP-listed characteristics would have exceeded practical limits on task length and respondent burden. This is a well-recognized constraint in conjoint survey design, reflecting both statistical concerns (e.g., non-orthoganality in the data) and practical concerns (e.g., participant fatigue) [19-22]. We therefore followed a standard set of test features or “attribute bundles” rather than the full list of TPP characteristics.

**[1] MedAccess. Viral load testing case study [Internet]. London: MedAccess; 2024 May 20 [cited 2026 Jul 28]. Available from:** [**https://medaccess.org/wp-content/uploads/2024/05/MedAccess_Viral-load-testing-case-study.pdf**](https://medaccess.org/wp-content/uploads/2024/05/MedAccess_Viral-load-testing-case-study.pdf)
